# Supplementary material for: MetaRibo-Seq measures translation in microbiomes
Source: Nat Commun. 2020 Jun 29;11:3268. doi: 10.1038/s41467-020-17081-z (PMC7324362; doi:10.1038/s41467-020-17081-z)
Supplement: Supplementary file 10 — Supplementary Data 7 [file 41467_2020_17081_MOESM10_ESM.zip › File2/Confidence_High_Taxonomy/115157_out.krona.html]

Javascript must be enabled to view this page.

members
magnitude
magnitudeUnassigned
count
unassigned
taxon
rank

115157\_out

2

2
2
superkingdom

2
1239
phylum

186801
2
class

order
2
186802

family
543314
1

1951595
1

SRS019068\_contig\_number\_36955
species

1
541000
family

species

SRS053398\_contig\_number\_contig-100\_37279.37279
1898205
1
